# Preparation of Hydrophobic Modified Silica with Si69 and Its Reinforcing Mechanical Properties in Natural Rubber

**DOI:** 10.3390/ma17133131

**Published:** 2024-06-26

**Authors:** Bo You, Shengming Jin

**Affiliations:** School of Resource Processing and Bioengineering, Central South University, Changsha 410083, China; 205612084@csu.edu.cn

**Keywords:** Si69, surface modification, dispersibility, silica/natural rubber composites, reinforcing property

## Abstract

The inherent large number of hydroxyl groups of silica poses strong hydrophilicity, resulting in poor dispersibility in the natural rubber matrix. Here, the silica’s surface was hydrophobically modified with [3-(triethoxysiliconyl) propyl] tetrasulfide (Si69) to improve the dispersibility and reinforce the mechanical properties of silica/natural rubber composites. The structure and morphology of modified silica were characterized by Fourier infrared spectroscopy (FTIR), thermogravimetric analysis (TGA), X-ray electron spectroscopy (XPS), nuclear magnetic resonance spectroscopy and the contact angle. Further, the mechanical properties, dynamic mechanical properties and morphology of silica/natural rubber composites were studied with a universal electronic tension machine, dynamic thermal mechanical properties analyzer (DMA) and scanning electron microscope (SEM). The experimental results show that the Si69 was successfully grafted onto the surface of silica, thereby significantly improving the water contact angle (a 158.6% increase) and enhancing the mechanical properties of modified silica/natural rubber composites.

## 1. Introduction

In recent years, the escalating demand for low-energy consumption and a green economy has spurred the swift progression of green tires with low rolling resistance [1,2] and excellent anti-skid [3,4] performance. The utilization of economically viable and environmentally benign nano-silica [5,6,7], as an alternative to traditional carbon black [8,9], is an attractive strategy for enhancing mechanical properties [10]. During the manufacturing process of green tires [11,12,13], a significant amount of silica fillers is often necessitated. However, the nano-silica widely used as filler exhibits high surface activity due to the surface’s bound hydroxyl groups [14,15], resulting in strong polarity and poor dispersibility. The poor dispersibility and compatibility between highly polar silica and a non-polar rubber matrix usually significantly restrict the reinforcing property and efficiency [16,17]. Hence, the need to improve the dispersibility of silica [18] is undeniable for their broader application.

The silane coupling agent has been employed to modify the surface of nano-silica [19,20], which can effectively diminish the abundance of hydroxyl groups present on the surface and enhance its dispersion within natural rubber materials [21,22,23]. This modification significantly improves the interfacing compatibility between nano-silica and the natural rubber matrix [24,25,26]. Among the commonly utilized coupling agents for modification, polythiosilane [27,28,29] stands out, encompassing compounds such as bis[3-(triethoxysilyl) propyl] tetrasulfide (Si69), bis[3-(triethoxysilyl) propyl] disulfide and γ-mercaptopropyl trimethoxysilane. During the vulcanization process, the sulfur atoms present in polythiosilane participate in cross-linking reactions with the polymer chain [30,31]. Among these compounds, Si69 is a widely acclaimed polysulfide silane coupling agent. The silicon hydroxyl groups on the hydrolysate of Si69 undergo dehydration and condensation reactions [32,33] with the silicon hydroxyl groups residing on the surface of the particles, thereby enhancing the surface compatibility of nano-silica. Furthermore, during the vulcanization of natural rubber, the sulfur atoms in Si69 cross-link with the polymer chain [34], forming a robust network structure that improves the mechanical properties [35,36] of the natural rubber materials.

In this study, Si69 was employed as a modifier for the wet modification of silica powder. After post-hydrolysis of Si69, the resulting hydrophobic groups underwent organic integration with the hydroxyl groups present on the surface of silica filler. This chemical interaction effectively transformed the hydrophilic nature of the silica particles into a hydrophobic state, thereby enhancing their dispersion within the natural rubber matrix. Furthermore, the mechanical properties of the natural rubber composites were significantly bolstered through the modification of silica filler, while their anti-skid capabilities exhibited notable improvements. This methodology involved conducting pilot tests on the production line of Fujian Sanming Zhengyuan Chemical Industry Co., Ltd. (Sanming, China) and successfully produced 800 tons of modified silica filler, converting the traditional dry modification process into a wet modification process and improving the modification efficiency. The products obtained through wet modification have been applied to rubber products, improving the mechanical properties of rubber. At this stage, it has been successfully implemented and created a certain economic value.

## 2. Materials and Methods

### 2.1. Materials

Natural rubber with vulcanization promoters of zinc carbonate and diphenyl guanidine (CZ and DPG, respectively), silica slurry (solid content of 20%), stearic acid, antioxidant 4020, zinc oxide, accelerator and sulfur were all obtained from Fujian Sanming Zhengyuan Chemical Co., Ltd. (Sanming, China). Sulfuric acid (analytically pure) was purchased from Aladdin Biochemical Technology Co., Ltd. (Shanghai, China). Ethanol (analytically pure) was supplied by Aladdin Biochemical Technology Co., Ltd. Si69 was purchased from Nanjing Shuguang Chemical Group Co., Ltd. (Nanjing, China).

### 2.2. Preparation of Modified Silica (MS-SiO_2_)

To prepare the modified silica powder, initially, 30 mL of deionized water was added to 300 g of silica slurry with a solid content of 20%. The mixture was vigorously stirred until a homogeneous liquid state was achieved. Subsequently, the slurry was transferred to a water bath and preheated to 60 °C. A mixture of Si69, deionized water, and anhydrous ethanol was prepared and gradually added to the silica slurry. The mixture was stirred continuously for 2 h to ensure thorough mixing.

Afterward, the pH of the slurry was adjusted to 4 by adding a precisely measured amount of dilute sulfuric acid. The slurry was then allowed to undergo a hydrolysis reaction until completion. Following the completion of the reaction, the resulting slurry was filtered to separate the solids.

**Figure 1:**
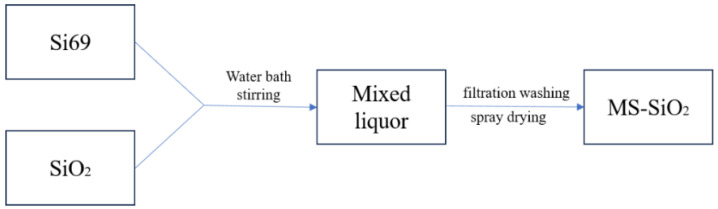
Process flow of surface modified silica.

The modified silica powder was subsequently washed thoroughly with anhydrous ethanol to remove any impurities. Finally, the washed silica powder was dried in a vacuum oven at 105 °C for 4 h to remove residual solvents and moisture, ensuring the final product’s purity and stability. The production process of modified silica is shown in Figure 1.

### 2.3. Preparation of SiO_2_/NR and MS-SiO_2_/NR Composites

The silica/natural rubber composites with excellent properties were prepared by mixing modified silica with natural rubber. In the first stage, the mold temperature of the two-roll smelting machine was set to 55 °C. Subsequently, the rubber used in the experiment was added to the two-roll smelting machine and mixed for 3 min at a speed of 60 r/min initially. To optimize the mixing process, the rotational speed of the two-roll open mill was reduced and adjusted to 15 r/min. Afterward, the modified silica was introduced into the mill in three separate additions, with each addition occurring at an interval of 1 min. Immediately following the addition of the modified silica, the antioxidant 4020, zinc oxide, accelerator, sulfur and other necessary additives were also incorporated into the mix. Finally, the rotational speed of the mill was adjusted to 60 r/min to facilitate thorough mixing and ensure homogeneity in the compounded material. The mixing process was continued for 50 min, during which the rubber sheet was repeatedly folded into a triangular package to guarantee thorough mixing. Once the mixing was completed, the resulting film was vulcanized on a flat vulcanizer to yield the composite material of silica and rubber. The vulcanized material was then allowed to sit at room temperature for 12 h to stabilize. Following this, a 5 g rubber sample was cut and subjected to vulcanization testing to determine the optimal vulcanization time. In accordance with standard procedures, the vulcanized rubber was subsequently preprocessed into the required shape for subsequent testing, including tensile strength, wear resistance and other physical property characterizations.

### 2.4. Characterization

The infrared spectrum of the material was analyzed using a Thermo Fisher Scientific (Waltham, MA, USA) (Nicolet iS50) spectrometer. Prior to analysis, the sample was thoroughly mixed with KBr, ground and compacted. The test parameters were set to 64 scanning runs with a resolution of 1 cm^−1^. Thermogravimetric analysis (TGA) was carried out using a NETZSCH STA 449 F3 (Germany Nechi instrument manufacturing Co., Ltd., Shanghai, China) Jupiter thermal analyzer, operating within a temperature range of 30–1000 °C and heating rate of 10 °C·min^−1^ in a nitrogen atmosphere at a flow rate of 200 mL/min.

To observe the dispersion differences in SiO_2_ before and after modification, an S4800 scanning electron microscope (Hitachi, Hitachi, Japan) was employed. Additionally, a video contact angle tester (JY-82C, Chengde Dingsheng Co., Ltd., Chengde, China) was used to determine the change in the sample’s contact angle.

To characterize the changes in the Si unit structure, a Bruker spectrometer (Leipzig, Germany) was utilized. The mechanical properties of the composite materials were tested using a universal electronic tensile machine (CMT4104, Shenzhen Si Measurement Technology Co., Ltd., Shenzhen, China). Furthermore, the dynamic mechanical properties of the natural rubber composites were tested using a dynamic thermomechanical analyzer (DMA) in the temperature range from −80 °C to 40 °C at a heating rate of 3 °C∙min^−1^ and a frequency of 12 Hz under tensile mode.

For the Mooney viscosity test, we collect a 4 g sample of natural rubber composite material and carefully positioned it in the mold of a Mooney viscometer. We proceeded by heating the mold and the sample to 100 °C. Once the desired test temperature was reached, this temperature was maintained for a sufficient duration to ensure uniformity throughout the sample. Subsequently, we inserted the rotor of the Mooney viscometer into the sample and activated the instrument. The test involved measuring the force required to rotate the rotor through a specific angle over a duration of 4 min.

To test the binding adhesive content [37,38], we took 1 g of the sample and dissolved it in 50 milliliters of toluene. We ensured the stirring solution was covered and agitated using magnetic force at room temperature for a duration of 48 h. Following this, the insoluble substances were separated from the solution by decanting. We then centrifuged the remaining solution to remove any precipitates and placed the resulting liquid in an oven set to 50 °C to dry until a constant weight was achieved. Finally, we determined the binding adhesive content of the composite material using the appropriate formula:

$$B\left(\%\right)=\frac{m_{0}-\left(m_{2}-m_{3}\right)}{m_{0}}\times 100\%\;m_{0}=\frac{m_{2}-m_{1}}{M_{compound}}\times 100\%$$

where $B\left(\%\right)$ is the percentage of bound rubber and *m*_0_, *m*_1_, *m*_2_, *m*_3_ and *M_ompound_* are the mass of the rubber in the initial sample, weight of the tube, weight of the tube plus the extracted sample, weight of the tube plus the extracted sample after drying process and the total formulation of the compound (in phr), respectively.

## 3. Results and Discussion

### 3.1. FTIR Analysis

**Figure 2:**
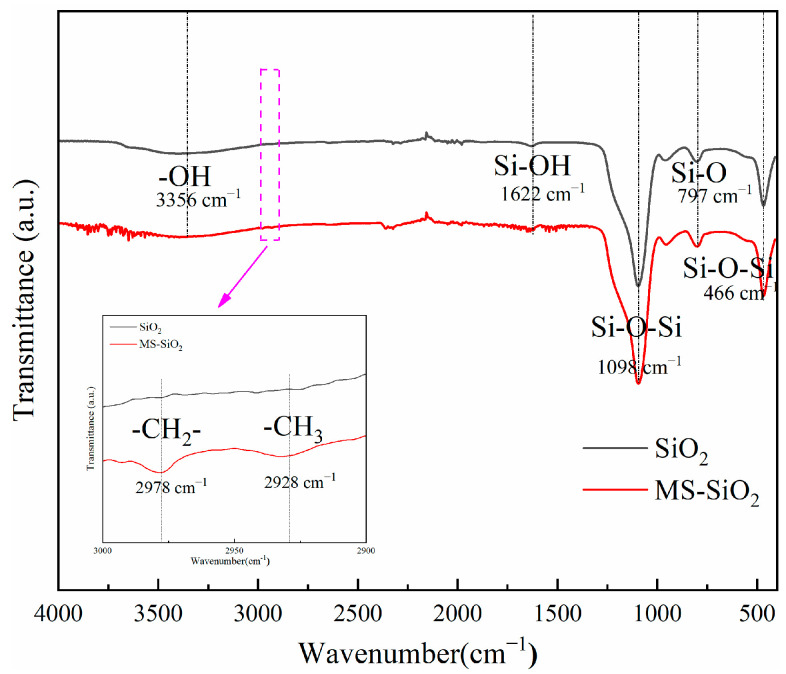
FTIR spectrum of silica before and after modification with Si69.

Figure 2 depicts the FTIR spectra of SiO_2_ and MS-SiO_2_. In the spectrum of SiO_2_, the antisymmetric stretching vibration peak of Si-O-Si was observed at 1098 cm^−1^, while the symmetric stretching vibration peak and bending vibration peak of Si-O-Si were located at 797 cm^−1^ and 466 cm^−1^, respectively.

Notably, it demonstrated a much weaker hydrogen bond absorption peak for adsorbed water on the surface of silica at 3356 cm^−1^. Additionally, an evident independent Si-OH vibration peak was observed at 3650 cm^−1^.In comparison, the FTIR spectrum of MS-SiO_2_ exhibited a distinct symmetric stretching vibration peak for -CH_2_- at 2978 cm^−1^ and an asymmetric stretching vibration peak for -CH_3_ at 2928 cm^−1^. These findings indicate successful grafting of the Si69 onto the surface of silica, confirming the modification process.

### 3.2. Thermal Decomposition Analysis

**Figure 3:**
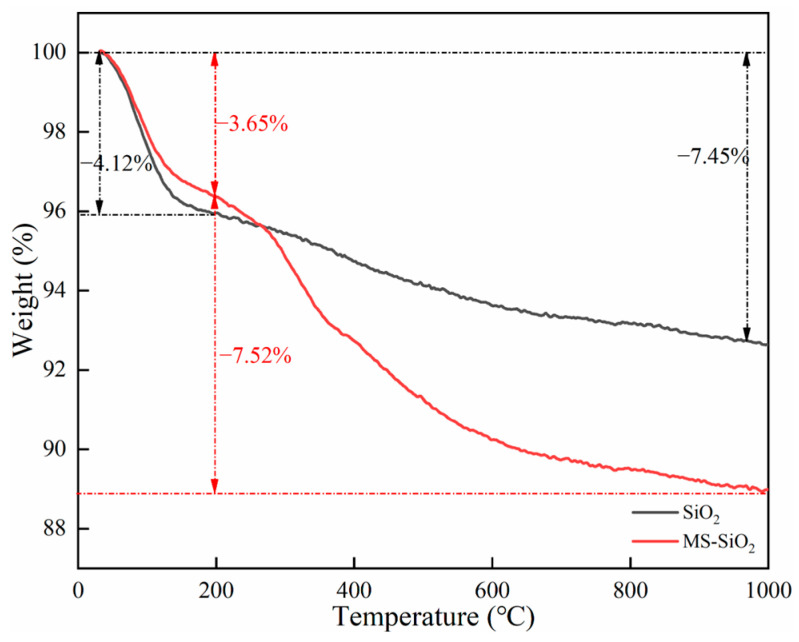
TG curves of silica before and after modification with Si69.

Figure 3 presents the thermogravimetric analysis (TGA) curves of SiO_2_ and MS-SiO_2_.

**Figure 4:**
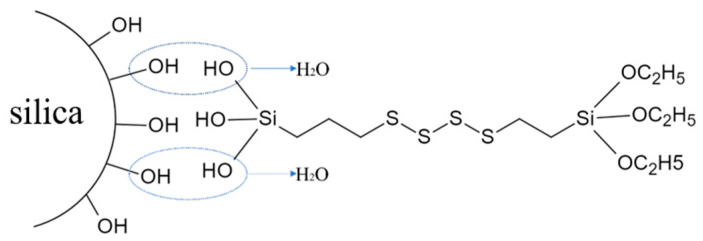
Reaction structure model between silica and Si69.

Meanwhile, Figure 4 illustrates the structural model representing the reaction between the silica and Si69.

As can be observed from the TG curves, within the temperature range from 30 °C to 200 °C, SiO_2_ and MS-SiO_2_ exhibited weight loss percentages of 4.12% and 3.15%, respectively. This weight loss was primarily attributed to the desorption of adsorbed water on the surface of SiO_2_. Notably, SiO_2_ exhibited a higher weight loss rate than MS-SiO_2_ in this range, indicating that the surface modification with Si69 may significantly reduce the adsorbed water content. With a higher temperature from 200 °C to 1000 °C, both SiO_2_ and MS-SiO_2_ exhibited gradual weight loss. For SiO_2_, this was primarily due to the condensation and dehydration of Si-OH groups on the surface. However, MS-SiO_2_ displayed a significantly higher weight loss from 250 °C to 390 °C. This was probably associated with the boiling point of Si69 being 250 °C, indicating incomplete hydrolysis of Si69. Additionally, the weight loss between 400 °C and 800 °C suggests decomposition of the chemically grafted -C3H6-S4-C3H6- groups on the silica surface at high temperatures, leading to gradual weight loss. This result is highly consistent with the FTIR analysis, further confirming the successful surface modification of the silica.

### 3.3. XPS Analysis

**Figure 5:**
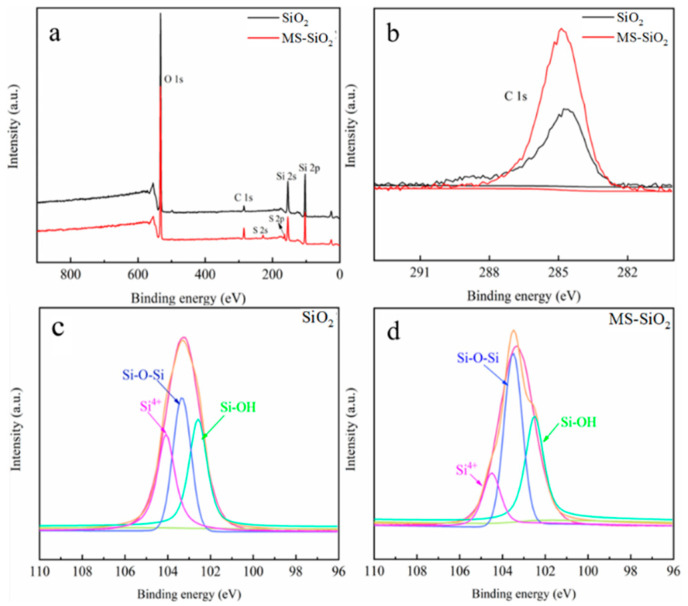
XPS diagram of silica before and after modification with Si69. (**a**): Full spectrum of silica; (**b**): Amplified spectrum of (**c**) element; (**c**,**d**) is Si2p peak fitting spectra.

XPS spectra were further used to analyze the chemical structures of SiO_2_ and MS-SiO_2_. As Figure 5a shows, both SiO_2_ and MS-SiO_2_ revealed the binding energies of Si2p, Si2s, C1s and O1s to be 103.28 eV, 154.32 eV, 284.67 eV and 532.67 eV, respectively.

Notably, the XPS full spectrum of MS-SiO_2_ exhibited S2p and S2s peaks at 163.95 eV and 228.18 eV, indicating the presence of the S element originating from Si69. Figure 5b displays the C1s peaking spectra of SiO_2_ and MS-SiO_2_. The concentration of the C element in SiO_2_ stood at 2.33%, while the C element concentration in MS-SiO_2_ reached 6.15%, demonstrating a significant increase of 264%. The MS-SiO_2_ C1s spectrum was primarily composed of C-O and C-H bonds, whereas the C element in the SiO_2_ was primarily in its elemental form.

Figure 5c,d shows the peak partial spectra of Si2p for SiO_2_ and MS-SiO_2_. A careful analysis of these spectra revealed that the intensity of the Si-O-Si peak (at 103.51 eV) in MS-SiO_2_ was notably higher than that in SiO_2_. This enhancement was attributed to the dehydration condensation of Si69 on the silica’s surface, leading to the formation of Si-O-Si bonds and the successful grafting of Si69.

### 3.4. ^29^Si NMR Spectrum Analysis

**Figure 6:**
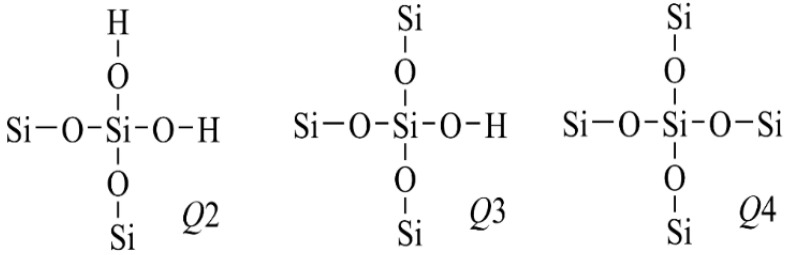
Three different structures of silicon structural units.

The ^29^Si NMR spectrum is usually used to characterize the structure of silicon compounds. As shown in Figure 6, there existed three distinct silica structural units with varying configurations before and after being modified with Si69.

**Figure 7:**
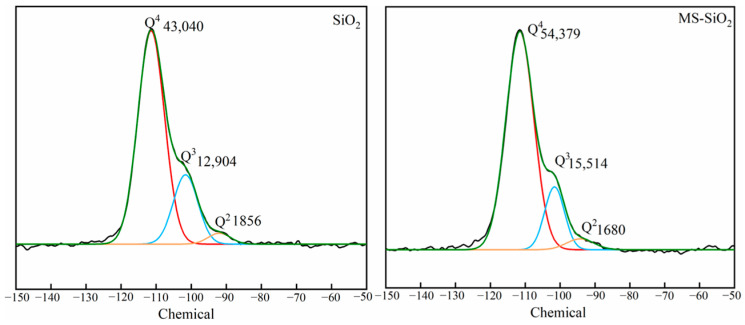
NMR before and after modification with silica.

Specifically, the chemical shifts at −92.2 ppm, −101 ppm and −110 ppm corresponded to the Q^2^(Si(2SiO)2OH), Q^3^(Si(3SiO)OH), and Q^4^(Si(4SiO)) structures, respectively. The newly introduced Si69 exhibited a homing peak in the ^29^Si NMR spectrum within the range from −55 ppm to −70 ppm. The peak-splitting fitting diagram of the ^29^Si NMR spectrum of silica black are presented in Figure 7, respectively.

After modification, the peak area ratio of Q2/Q4 decreased from 4.31% to 3.08%, indicating a reduction in Si(-OH)_2_ on the surface of the silica due to the reaction with Si69.

Furthermore, the peak area ratio of Q3/Q4 in the silica modified with Si69 decreased from 29.98% to 28.53%. Given that the hydrolyzed Si69 terminates in three silicon hydroxyl groups, it is challenging for all three groups to simultaneously react with the three silicon hydroxyl groups present on the surface of silica. However, the similar decline rates of Q2/Q4 and Q3/Q4 suggest that the hydroxyl groups on the surface of Si69 reacted completely. This indicates that Si69 primarily reacts with the hydroxyl groups, and the terminal hydroxyl groups of grafted Si69 also undergo polymerization, leading to the formation of a spatial network structure.

### 3.5. Surface Wettability of SiO_2_ and MS-SiO_2_

**Figure 8:**
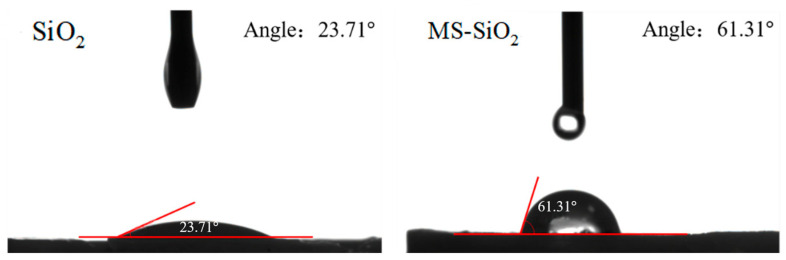
Change in contact angle of silica before and after being modified with Si69.

Figure 8 exhibits the water contact angles of both the SiO_2_ and MS-SiO_2_ surfaces.

Once in contact with the SiO_2_ surface, water droplets were rapidly absorbed, resulting in a contact angle of 23.71°. This absorption occurred due to the large voids between the silica particles and the abundant hydrophilic Si-OH groups present on the silica surface, which facilitated capillary action between the particles. In contrast, after being modified with Si69, the contact angle of MS-SiO_2_ increased significantly. This observation suggests a substantial reduction in the number of hydrophilic Si-OH groups on the silica black surface, leading to an enhancement in the hydrophobicity of the silica powder. Specifically, the contact angle of silica powder modified with Si69 reached 61.31°, representing a 158.6% increase compared with that of SiO_2_. This substantial increase in the contact angle unequivocally demonstrated the improved hydrophobicity of the silica black powder achieved through modification with Si69.

### 3.6. The Micromorphology of SiO_2_ and MS-SiO_2_

**Figure 9:**
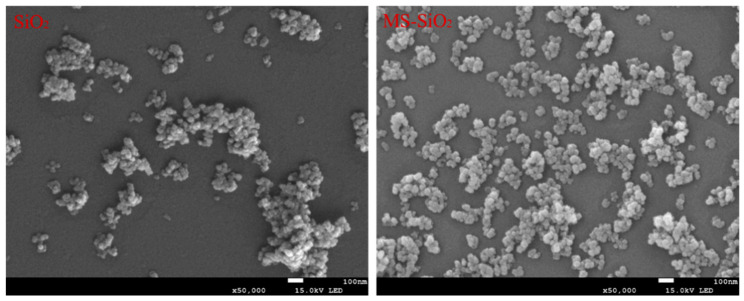
Microscopic morphology of silica before and after being modified with Si69.

The micromorphology of SiO_2_ and MS-SiO_2_ was investigated with SEM tests. As shown in Figure 9, the SiO_2_ exhibited an apparent agglomeration phenomenon, characterized by a substantial clustering of particles and exceedingly poor dispersion.

A comparative analysis of the SEM images revealed a significantly reduced aggregation tendency in the silica particles after being modified with Si69, leading to improved dispersibility. The underlying reason for this observation was the decrease in the hydroxyl groups on the surface of the silica. This reduction mitigates the agglomeration phenomenon caused by the condensation of hydroxyl groups during the drying process, thereby significantly enhancing the dispersion of the modified silica powder.

### 3.7. Mechanical Properties of SiO_2_/NR and MS-SiO_2_/NR

**Figure 10:**
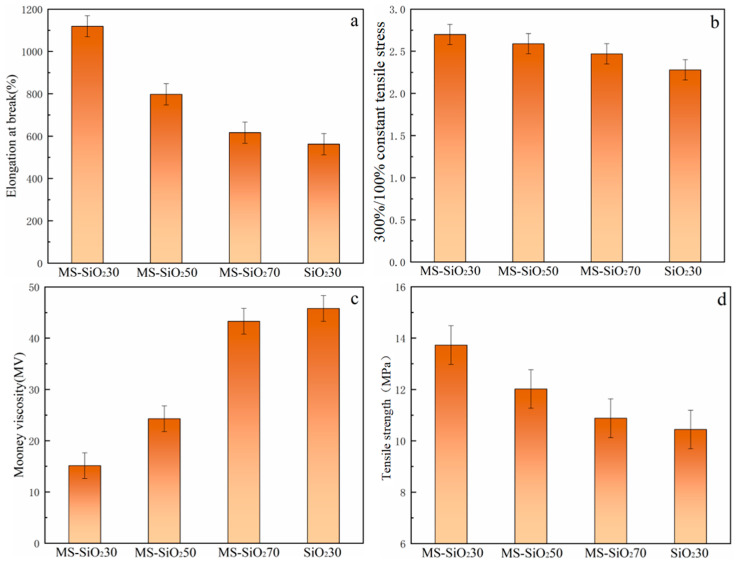
Mechanical properties of composite materials. (**a**): Elongation at break of composite materials; (**b**): 300% constant tensile strength of composite materials; (**c**): Mooney viscosity of composite materials; (**d**): Tensile stress of composite materials.

Figure 10 and Table 1 present the mechanical properties of the composites formulated by incorporating varying fractions of MS-SiO_2_ into natural rubber.

Compared with the mechanical properties of the pure NR and MS-SiO_2_/natural rubber composites, it can be seen that the addition of 30 phr MS-SiO_2_ to strengthen natural rubber could reduce the Mooney viscosities of the composite materials, the binding ability of MS-SiO_2_ particles to the rubber matrix was improved, the molecular fluidity was enhanced, and the elongation at break and constant elongation stress were increased. By comparing the mechanical properties of the pure NR and MS-SiO_2_/natural rubber composites, it can be seen that the addition of 30 phr MS-SiO_2_ to strengthen natural rubber could reduce the Mooney viscosities of the composite materials, and the binding ability of MS-SiO_2_ particles to the rubber matrix could be improved. The molecular fluidity increased, while the elongation at break and the constant elongation stress increased, indicating that adding MS-SiO_2_ to strengthen natural rubber can improve the mechanical properties of the composite. Compared with pure NR, the tear strength of the reinforced rubber composite decreased to a certain extent because the movement ability of the rubber molecular chain improved after the addition of the filler. The comparison of constant elongation stress values shows that the constant elongation stress of the composite material after adding filler was significantly higher than that of pure NR. Thus, the addition of SiO_2_ filler can improve the mechanical properties of natural rubber composites to a certain extent. Specifically, the elongation at break, the ratio between the 300% and 100% constant elongation stresses and the density of the composite achieved by incorporating 30 parts of MS-SiO_2_ into natural rubber exceeded those achieved with 50 and 70 parts. This trend can be attributed to the fact that as the number of silica fillers increased, the proportion of fillers within the natural rubber composite material rose accordingly. Consequently, the overall colloidal content of the composite diminished, leading to an augmentation in the formed filler network. This network, in turn, hindered the molecular chain slip in the natural rubber composite, causing a continuous rise in its Mooney viscosity. Notably, when the quantity of MS-SiO_2_ fillers surpassed 30 parts, a filling interference phenomenon emerged, resulting in a decrement in the reinforcing effectiveness of the silica fillers.

In detail (Figure 10a), the elongation at break of the natural rubber composites underwent a decrement with an increasing number of MS-SiO_2_ fillers.

This observation was attributed to the enhanced formation of a filler network by the excessive proportion of modified silica within the natural rubber. This augmentation of the network led to a deterioration in the bonding between the natural rubber matrix and the modified silica fillers. Consequently, the molecular chains within the natural rubber composite became more susceptible to slipping, resulting in enhanced vulnerability to fracturing and a consequent reduction in the elongation at break. On the contrary, Figure 10b illustrates that the ratio of 300% constant elongation stress to 100% constant elongation stress escalated with an increasing number of filler parts. This trend suggests that when the amount of modified silica filler is set to 30 parts, the adhesive interaction between the filler particles and natural rubber is optimized, leading to superior reinforcement performance. Therefore, it is evident that when the modified silica filler was present in a quantity of 30 parts, the reinforcing effect of the filler was optimal. As depicted in Figure 10c, the viscosity of the MS-SiO_2_-reinforced composite exhibited a substantial decrease, achieving a 66.9% reduction in its Mooney viscosity compared with when the same number of parts was used to reinforce natural rubber alone. Furthermore, as the number of filler parts gradually increased, the Mooney viscosity also rose, which was attributed to the uneven dispersion of MS-SiO_2_ within the composite. As shown in Figure 10d, the tensile strength decreased progressively with an increasing amount of MS-SiO_2_, suggesting an augmentation in the aggregation of reinforced particles within the rubber matrix.

### 3.8. Dynamic Thermomechanical Analysis of SiO_2_/NR and MS-SiO_2_/NR

**Figure 11:**
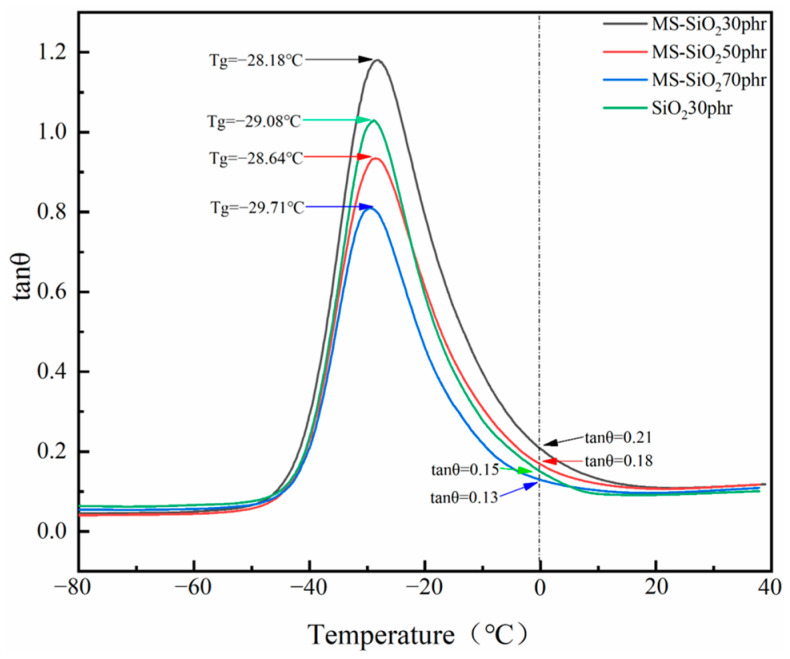
Loss factors of composite materials.

Tanθ, alternatively referred to as the loss angle or dissipation angle of a material, serves as a useful means to characterize the mechanical properties of polymer materials. Figure 11 shows the tanθ curve of the composite material formed by modified silica filler with 30, 50 and 70 parts and the composite material formed by unmodified SiO_2_ and natural rubber with varying temperatures. The tangent of the loss angle (tanθ = E″/E′) is the ratio between the loss modulus and the energy storage modulus in the process of constant stress change. This represents the degree of lag to the mechanical response. It can be seen from the figure that when the MS-SiO_2_30/NR composite material was added, the corresponding tanθ was the largest at a temperature Tg, which was due to the better dispersion of the MS-SiO_2_ filler in the natural rubber matrix. The filler could be evenly dispersed in the natural rubber matrix, the degree of binding between the filler and the natural rubber matrix increased, and the loss factor of the composite material increased. In the figure, it can be seen that the order of loss factors tanθ of the SiO_2_ natural rubber composite material modified at 0 °C was MS-SiO_2_30 > MS-SiO_2_50 > MS-SiO_2_70, and the loss factor of the rubber composite at 0 °C was related to the skid resistance. Thus, when the amount of modified silica was 30 parts, the natural rubber composite had the best skid resistance. As the modified silica continued to be added, the anti-skid performance of the composite decreased. This is because the interference distribution occurred when the filler was larger than 30 parts, a large number of silica particles gathered, the volume fraction of the filler increased, the dispersion decreased, and the degree of the cross-linked network formed by the rubber matrix on the modified silica decreased.

### 3.9. TG and DSC Analysis of NR, NR/SiO_2_30 and NR/MS-SiO_2_30

**Figure 12:**
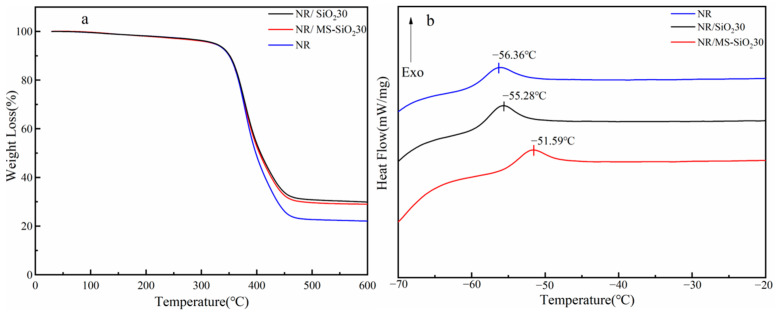
(**a**,**b**) is TG and DSC analysis of composites.

Figure 12 shows the TG change curves of different composites at 0–600 °C and the DSC change curves from −70 °C to −20 °C. For the Tg curve, it can be seen that the weight loss of the natural rubber composites with SiO_2_ and MS-SiO_2_ fillers was smaller than that of NR because the added fillers had high stability and did not decompose easily at high temperatures. The weight loss of the MS-SiO_2_/NR composites was slightly less than that of the SiO_2_/NR composites, which may be due to the fact that the organic groups grafted onto modified silica decompose at high temperatures, while pure silica does not exist in this stage. It can be seen from the DSC curve that the glass transition temperature of NR = −56.36 °C, the glass transition temperature of NR/SiO_2_30 = 55.28 °C, and the glass transition temperature of NR/MS-SiO_2_30 = −51.59 °C. The addition of filler can increase the Tg of natural rubber composite. The Tg of the composite formed by the addition of modified silica filled with natural rubber increased significantly, indicating that the combination degree of modified silica and the natural rubber matrix was higher, mainly because the sulfur chain of Si69 organic groups was conducive to the vulcanization of the composite, and the Tg of the composite formed by the addition of pure silica filled with natural rubber was also slightly higher than that of NR. This may be due to the hydrogen bonding between silica particles and the rubber matrix, which improved the fluidity of the rubber molecular chains, and this result is consistent with the mechanical properties.

### 3.10. Vulcanization Properties of SiO_2_/NR and MS-SiO_2_/NR

**Figure 13:**
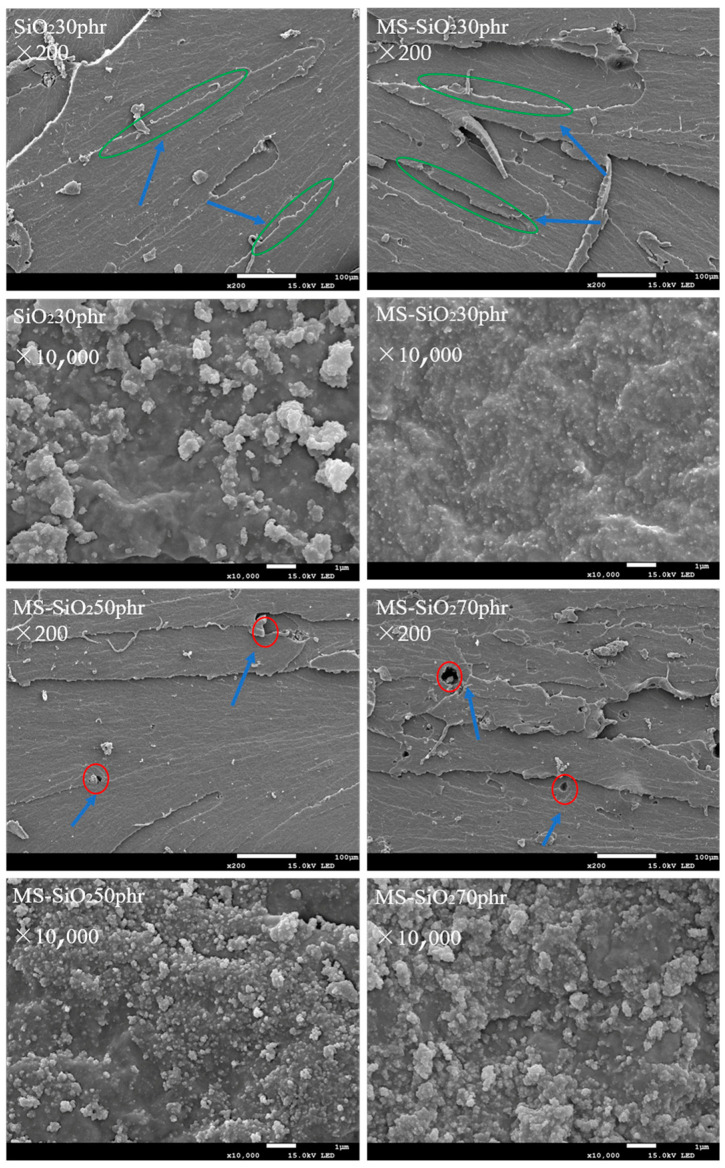
SEM of composite materials.

Figure 13 displayed the tensile fracture surfaces of the SiO_2_-reinforced natural rubber with filler at 30 phr and MS-SiO_2_-reinforced natural rubber with filler at 30 phr, 50 phr and 70 phr.

Notably, when SiO_2_ and MS-SiO_2_ were used to reinforce natural rubber at 30 phr, a significant increase in tensile cracks on the rubber fracture surface was observed, Green oval marks in the figure. This phenomenon may be attributed to the improved dispersion of modified silica particles and the enhanced interfacial adhesion between MS-SiO_2_ and the natural rubber matrix. This led to an increase in internal stress and an improvement in the mechanical properties of the rubber composite.

However, as the quantity of MS-SiO_2_ exceeded 30 phr, voids became apparent on the tensile fracture interface of the composite, The red circle is marked in the figure. Suggesting that excessive MS-SiO_2_ addition impairs the compatibility between MS-SiO_2_ and the natural rubber matrix, resulting in inferior dispersion. At higher magnifications, it was further evident that the addition of filler at more than 30 phr led to an increase in the aggregation of filler particles, thereby reducing the mechanical properties of the composite.

### 3.11. Binding Adhesive Content of NR, NR/SiO_2_30 and NR/MS-SiO_2_30 Composites

**Figure 14:**
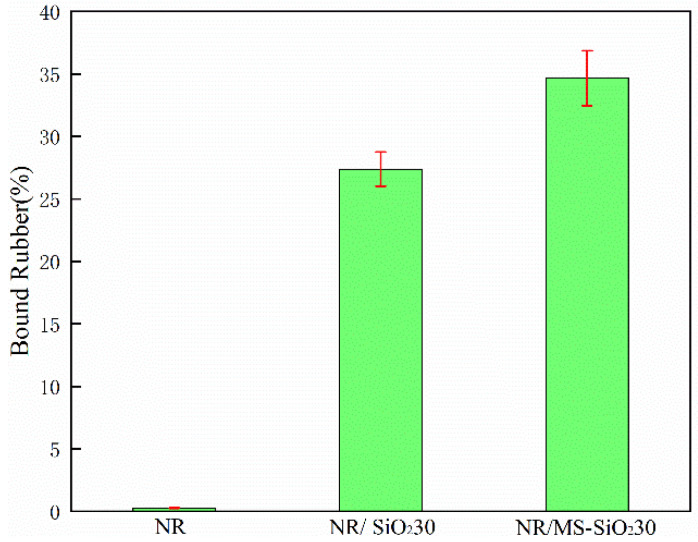
Binding glue content of composite materials.

Figure 14 shows the binding glue content of the composite material (i.e., the polymer that could not be extracted from the suspension by the solvent at room temperature), indicating the binding degree of the filler particles and the rubber matrix. The binding glue played an important role in the formation of the cross-linked network of natural rubber composite materials. It can be seen in the figure that pure NR had a small amount of binding glue, which have may been caused by magazines, and the binding amount of MS-SiO_2_ significantly increased after adding MS-SiO_2_, being 26.6% higher than that of the natural rubber composite with pure SiO_2_ filler. The dispersion of modified silica improved, and the binding degree with natural rubber improved. On the other hand, the Si69 organic groups introduced in the modification process contained sulfur chains that combined with rubber molecular chains to form a cross-linked network, which improved the binding rubber content of the composite material and thus improved the mechanical properties of the composite material.

## 4. Conclusions

Based on the comprehensive experimental investigation conducted, the following conclusions were drawn:

1. The wet modification process successfully grafted Si69 onto the surface of silica powder. FTIR spectrum analysis revealed the presence of distinct peaks corresponding to the -CH_3_ and -CH_2_ groups, indicating successful modification. This transformation converted the hydrophilic silica slurry into hydrophobic silica powder, resulting in a substantial increase in the contact angle of MS-SiO_2_ from 23.71° to 61.31°, representing a 158.6% augmentation. Furthermore, SEM analysis revealed that the Si69-modified silica powder exhibited superior dispersion characteristics.
2. The integration of MS-SiO_2_ with natural rubber to form a composite material significantly enhanced the mechanical properties of MS-SiO_2_/NR. Specifically, the elongation at break was augmented by 99%, while the Mooney viscosity was reduced by 66.97%. Notably, when MS-SiO_2_ at 30 phr was added, the tanθ value of the composite reached its peak of 0.21 at 0 °C, indicating optimal skid resistance properties. However, further incrementing the MS-SiO_2_ content for rubber reinforcement led to uneven dispersion of MS-SiO_2_, thereby adversely affecting the overall performance of the composite material.

## Figures and Tables

**Table 1:** Mechanical properties of composite materials.

Sample	NR	MS-SiO_2_ (30 phr)	MS-SiO_2_ (50 phr)	MS-SiO_2_ (70 phr)	SiO_2_ (30 phr)
Mooney viscosity	20.2	15.1	24.3	43.3	45.8
Tensile stress (MPa)	24.1	13.7	12.0	10.8	10.4
Elongation at break (%)	725.3	1119.3	797.7	616.7	562.5
100% constant tensile stress (MPa)	0.79	0.9	1.2	1.8	1.5
300% constant tensile strength (MPa)	1.82	2.5	3.3	4.5	3.4
500% constant tensile strength (MPa)	4.18	4.9	6.9	8.8	8.5
Tear stress (MPa)	27.51	19.2	23.1	26.5	26.1

## Data Availability

The original contributions presented in the study are included in the article, further inquiries can be directed to the corresponding author.

## References

[1] Chi C., Wang P., Qian W., Zhang Y., Chen Q. (2022). Cardanol grafted onto liquid isoprene rubber like groud brothers hanging on the vine: A green plasticizer and compatibilizer. Mater. Today Commun..

[2] Liu X., Zhou X., Yang C., Huang J., Kuang F., Wang H. (2020). Study on the effect of particle size and dispersion of SiO_2_ on tribological properties of nitrile rubber. Wear.

[3] Fu W., Wang L. (2016). Research on Payne effect of natural rubber reinforced by graft-modified silica. J. Appl. Polym. Sci..

[4] Li S., Luo Y., Yongjun C., Xu T., Zhong B., Jia Z., Jia D. (2019). Enhanced Mechanical and Processing Property of Styrene-butadiene Rubber Composites with Novel Silica-supported Reactive Processing Additive. Fibers Polym..

[5] Neena G., Amrutha S.R., Rani J., Jose P.M., Mathiazhagan A. (2021). Nano-silica as reinforcing filler in NR latex: Role of processing method on filler morphology inside the rubber and properties of the nanocomposite. Express Polym. Lett..

[6] Tian Q., Zhang C., Tang Y., Liu Y., Niu L., Ding T., Li X., Zhang Z. (2021). Preparation of hexamethyl disilazane-surface functionalized nano-silica by controlling surface chemistry and its “agglomeration-collapse” behavior in solution polymerized styrene butadiene rubber/butadiene rubber composites. Compos. Sci. Technol..

[7] Jayabalakrishnan D., Saravanan K., Ravi S., Prabhu P., Maridurai T., Prakash V.R.A. (2020). Fabrication and Characterization of Acrylonitrile Butadiene Rubber and Stitched E-Glass Fibre Tailored Nano-Silica Epoxy Resin Composite. Silicon.

[8] Pöschl M., Vašina M., Zádrapa P., Měřínská D., Žaludek M. (2020). Study of Carbon Black Types in SBR Rubber: Mechanical and Vibration Damping Properties. Materials.

[9] Chen J., Hu M., Li Y., Li R., Qing L. (2023). Significant Influence of Bound Rubber Thickness on the Rubber Reinforcement Effect. Polymers.

[10] Pang S., Yu Y., Zhang L., Wu Y. (2021). Adjusting silica/rubber interfacial interactions and properties via the click reactions between liquid polybutadiene and silane. Compos. Sci. Technol..

[11] Shoul B., Marfavi Y., Sadeghi B., Kowsari E., Sadeghi P., Ramakrishna S. (2022). Investigating the potential of sustainable use of green silica in the green tire industry: A review. Environ. Sci. Pollut. Res..

[12] Lolage M., Parida P., Chaskar M., Gupta A., Rautaray D. (2020). Green Silica: Industrially scalable & sustainable approach towards achieving improved “nano filler–Elastomer” interaction and reinforcement in tire tread compounds. Sustain. Mater. Technol..

[13] Zheng X., Song S.K., Zhou Z., Jiang X., Sui Y., Che M., Xu Q., Wang Y., Zhao S., Li L. (2022). Effect of silica dispersed by special dispersing agents with green strategy on tire rolling resistance and energy consumption. J. Appl. Polym. Sci..

[14] Ma Q., Zheng L., Zhang G., Shang C., Fang H. (2020). Numerical modeling of sintering and dehydroxylation of porous silica preform for low-hydroxyl silica glass fabrication. Ceram. Int..

[15] Wang B., Hu J., Liu K., Zhang L., Jiang H., Li C. (2023). Reinforcement mechanism of silica surface hydroxyl: The opposite effect. Appl. Surf. Sci..

[16] Linec M., Mušič B. (2019). The Effects of Silica-Based Fillers on the Properties of Epoxy Molding Compounds. Materials.

[17] Jong L. (2020). Synergistic Effect of Calcium Carbonate and Biobased Particles for Rubber Reinforcement and Comparison to Silica Reinforced Rubber. J. Compos. Sci..

[18] Gashti M.P., Moradian S., Rashidi A., Yazdanshenas M.-E. (2013). Various nano-silica particles affecting dyeability of poly(ethylene terephthalate)/silica nanocomposite films. Fibers Polym..

[19] Wang X., Zhang C., Wu Q., Zhu H., Liu Y. (2021). Thermal properties of metakaolin-based geopolymer modified by the silane coupling agent. Mater. Chem. Phys..

[20] Rong Z., Zhao M., Wang Y. (2020). Effects of Modified Nano-SiO_2_ Particles on Properties of High-Performance Cement-Based Composites. Materials.

[21] Kumkrong N., Dittanet P., Saeoui P., Loykulnant S., Prapainainar P. (2022). Properties of silica/natural rubber composite film and foam: Effects of silica content and sulfur vulcanization system. J. Polym. Res..

[22] Nuinu P., Sirisinha C., Suchiva K., Daniel P., Phinyocheep P. (2023). Improvement of mechanical and dynamic properties of high silica filled epoxide functionalized natural rubber. J. Mater. Res. Technol..

[23] Olejnik O., Masek A. (2022). Natural Phenolic Compounds as Modifiers for Epoxidized Natural Rubber/Silica Hybrids. Molecules.

[24] Li M., Wang K., Xiong Y. (2021). Multiple Intermolecular Interaction to Improve the Abrasion Resistance and Wet Skid Resistance of Eucommia Ulmoides Gum/Styrene Butadiene Rubber Composite. Materials.

[25] Saengdee L., Pasetto P., Sukhawipat N. (2024). Functionalization of mesoporous silica with oligoisoprene brushes: Application as charge for elastomers reinforcement. Prog. Org. Coat..

[26] Thi T.N., Van H.D., Hong H.C., Ha H.N.T., Hayati Y.N., Kawahara S. (2021). Preparation and properties of colloidal silica-filled natural rubber grafted with poly(methyl methacrylate). Polym. Bull..

[27] Tian Q., Tang Y., Ding T., Li X., Zhang Z. (2018). Effect of nano-silica surface-capped by bis[3-(triethoxysilyl)propyl] tetrasulfide on the mechanical properties of styrene-butadiene rubber/butadiene rubber nanocomposites. Compos. Commun..

[28] Losev V.N., Borodina E.V., Buyko O.V., Samoilo A.S., Elsuf‘Ev E., Li M. (2023). Highly selective adsorbents based on silica gel chemically modified with sulfur-containing groups of arched structure for preconcentration and determination of palladium(II) in products of processing of sulfide copper-nickel ore. Microchem. J..

[29] Jin X., Wu X., Cui S., Wang W., Zhang Y., Sun S., Sun D. (2022). A robust adhesion between extruded polystyrene foam and mortar through different chemical linkages under ultraviolet-ozone irradiation. Constr. Build. Mater..

[30] Jalkanen T. (2021). Isoconversional kinetic analysis for determining the rate of cross-linking for Pt and peroxide cure silicone rubbers. Thermochim. Acta.

[31] Weißer D.F., Mayer D., Schmid J., Deckert M.H. (2022). Novel approach to characterize the cross-linking effect of liquid silicone rubber via cavity pressure analysis during injection molding. J. Appl. Polym. Sci..

[32] Zhang J., Lu P., Teng Y., Wang S., Zhang L. (2021). In-situ surface modification of precipitated silica nanoparticles with 3-methacryloxypropyltrimethoxysilane in carbonation process. Res. Chem. Intermed..

[33] Xu Z., Ye Y., Tang G., Liu Y., Yang R. (2023). Effect of alkylated surface modified silica nanoparticles on degradation products of PP during photooxidation aging: A Py-GC-MS analysis. J. Anal. Appl. Pyrolysis.

[34] Alam N., Kumar V., Debnath S.C., Jeong T., Park S.-S. (2023). Naturally abundant silica-kaolinite mixed minerals as an outstanding reinforcing filler for the advancement of natural rubber composites. J. Polym. Res..

[35] Meng Z., Li J., Zou Y., Li N., Fu X., Zhang R., Hu S., Liu Q. (2022). Advanced montmorillonite modification by using corrosive microorganisms as an alternative filler to reinforce natural rubber. Appl. Clay Sci..

[36] Adibi A., Simon L., Lenges C., Mekonnen T.H. (2023). Sustainable natural rubber composites: Masterbatch development of epoxidized natural rubber grafted to designed enzymatic polysaccharides. Mater. Chem. Front..

[37] Bach Q.-V., Vu C.M., Vu H.T. (2019). Effects of Co-Silanized Silica on the Mechanical Properties and Thermal Characteristics of Natural Rubber/Styrene-Butadiene Rubber Blend. Silicon.

[38] Chonkaew W., Minghvanish W., Kungliean U., Rochanawipart N., Brostow W. (2011). Vulcanization Characteristics and Dynamic Mechanical Behavior of Natural Rubber Reinforced with Silane Modified Silica. J. Nanosci. Nanotechnol..

